# Response of healthy local tomato (*Solanum lycopersicum* L.) populations to grafting in organic farming

**DOI:** 10.1038/s41598-019-41018-2

**Published:** 2019-03-14

**Authors:** Marta María Moreno, Jaime Villena, Sara González-Mora, Carmen Moreno

**Affiliations:** University of Castilla-La Mancha, Technical School of Agricultural Engineering in Ciudad Real, Ronda de Calatrava 7, 13071 Ciudad Real, Spain

## Abstract

Demands for tomato local varieties are increasing worldwide, especially in organic farming mainly for their high sensory value and attractive appearance. This is the case of the “Moruno” tomato type, widely grown in the Mediterranean countries and greatly due to its highly appreciated organoleptic attributes but low yield or a short postharvest period. For this reason, the study aimed to assess if grafting of local “Moruno” populations (Mor-62, Mor-204) using commercial rootstocks (King-Kong F1, K; Multifort F1, M; Spirit F1, S) affects yield, fruit nutritional and functional composition, postharvest storage and consumer acceptance. Results showed differences between both populations, while rootstocks were only different for the glucose content and the flavour quality. Grafting improved the marketable yield (~43%), fruit number (~22%) and mean fruit weight (~12%), but had no incidence on the blossom-end rot disorder. This technique increased the carotenoid (lycopene, β-carotene and total carotene) content but decreased the organic acids (malic and citric) and sugar (fructose and glucose) rates, while ascorbic acid was not affected. The fruit postharvest storage was not practically modified. However, the overall flavour preference and visual appearance varied depending on the scion.

## Introduction

Traditional varieties of many cultivated species have been replaced by modern bred cultivars that are normally more productive and more resistant to diseases and pests^1^, resulting in a decline in the broad diversity of organoleptic and nutritional quality characteristics^2^. However, in recent years, consumer demand for fresh tomato (*Solanum lycopersicum* L.) fruits obtained from local varieties is increasing considerably worldwide, mainly for their high sensory value and their attractive appearance^3^. Additionally, local varieties are better adapted to specific agroclimatic conditions, and are therefore especially recommended for organic production^1^, a sustainable and low input agriculture. One of the most cultivated tomato landraces in different mountain areas from Mediterranean countries is the “Moruno” type, a savoury fruit much appreciated by consumers for its dark colour and highly appreciated organoleptic attributes, but with a short postharvest period of fruits, or low yield in many populations. However, in previous works on the “Cuore di Bue” heirloom tomato, this latter problem was solved by the use of grafting^4^.

Grafting is the union of two or more pieces of living plant tissue that grow as a single plant, after a complex process of formation of the connective structure between the rootstock and scion^5^. It was used in the past to limit the effects of soil-borne diseases in successive cropping^6^. However, the reasons for grafting as well as the kinds of vegetable grafted have increased considerably over the years. A detailed review about the general effect of this technique on vegetable crops can be consulted in recent reports^7–9^.

In the tomato, one of the most important horticultural crops in the world, the effect of grafting has also been widely studied. In general, these effects can be summarized as limiting the effects of soil-borne diseases, increasing plant vigour and crop yield under normal growing conditions^6^, or inducing tolerance to abiotic stresses^10–12^. However, in relation to the effect of grafting on tomato fruit quality, the results are contradictory^13^. The differences in reported results may be attributable in part to different production environments and methods, type of the rootstock/scion combination used, and harvest date^5,6^. The review developed by Davis *et al*.^14^ shows that in general sweetness decreases and carotenoids increase in grafted tomato fruits. However, different results about the nutritional and functional composition of fruits can be consulted in previous studies^4,15–17^.

Among the different components of tomato fruits, the antioxidant group composed of carotenoids (lycopene, β-carotene and total carotene) and ascorbic acid are very important for human health and crucial to the nutritional and functional value of tomato fruit, and due to the high consumption rates of these vegetables in our diet, they can provide a significant part of the total intake of these components^17^.

Parallel, it is important to examine the possible impact of grafting on consumer acceptance and overall organoleptic features, because consumption by the markets will depend largely on these things. In relation to this, only a few studies have analysed the effect of grafting on the sensory properties of tomato fruits^4^. To the best of our knowledge, no information is available about how grafting affects the postharvest period of stored fruits, which is very important for producer and consumer satisfaction, especially for traditional cultivars.

A further point is that most studies have been carried out on commercial tomato varieties. However, the response of local varieties, in general not adapted to exotic diseases, can be different and specific for each one, and would be interesting to enable their safe cultivation and to improve their production levels and fruit postharvest period without affecting the fruit quality traits. In this sense, only a few studies focused on the cultivars Brandywine and Flamme^18^ and on the “Cuore di Bue” heirloom tomato^4,16,17^, have been found.

Therefore, the aim of this study was to assess whether grafting onto commercial rootstocks is a valid technique to improve yield and fruit postharvest period of two traditional populations of the “Moruno” tomato type, widely appreciated in organic markets, as well as its possible effect on fruit nutritional and functional composition and on sensory traits.

## Results

### Yields and yield components

The comparison of the two ungrafted populations of the “Moruno” tomato type (Mor-62 and Mor-204) indicates that Mor-204 had a 70% and a 94% higher marketable and total fruit number than Mor-62, respectively, although the mean marketable fruit weight was 45% lower (Table 1). However, no significant differences in marketable and total yields were found (average values of 7.39 and 7.73 kg pl^−1^, respectively). The incidence of the blossom-end rot (BER) fruit disorder  was about 10 times higher in Mor-204.

**Table 1:** Effect of population, rootstock and grafting on yield variables of “Moruno” tomato type.

Treatment	Marketable yield			BER yield	Total yield	
	kg·plant^−1^	Fruit·plant^−1^	Fruit weight (g)	kg·plant^−1^	kg·plant^−1^	Fruit·plant^−1^
**Ungrafted**						
Mor-62	7.05 a B	22.2 b B	317 a A	0.06 b A	8.41 a B	27.2 b B
Mor-204	6.49 a B	37.7 a B	173 b A	0.59 a A	8.28 a B	52.8 a A
Mean	6.77 $	29.9	245	0.33	8.34$	40.0
**Grafted**						
***Main effects ANOVA***						
Population						
Mor-62	10.84 a A	31.5 b A	349 a A	0.10 b A	13.2 a A	40.6 b A
Mor-204	8.58 b A	41.8 a A	203 b A	0.71 a A	11.0 b A	56.3 a A
Mean	9.71 $	36.6	276	0.40	12.1$	48.5
Rootstock						
King-Kong (K)	10.95	42.4	266	0.32	13.7	53.7
Multifort (M)	9.40	34.5	289	0.43	11.3	45.0
Spirit (S)	8.79	32.9	274	0.46	11.3	46.7
*Significance*						
Population (P)	*	*	*	*	*	*
Rootstock (R)	NS	NS	NS	NS	NS	NS
P × R	NS	NS	NS	NS	NS	NS
Mor-62						
King-Kong (K)	12.07	36.0	335	0.05	14.3	44.8
Multifort (M)	10.35	27.6	375	0.08	12.2	33.6
Spirit (S)	10.11	30.8	338	0.15	13.3	43.4
Mor-204						
King-Kong (K)	9.83	48.8	197	0.58	13.2	62.6
Multifort (M)	8.44	41.7	203	0.78	10.4	56.4
Spirit (S)	7.46	35.0	210	0.76	9.36	50.0

NS: not significant; *significance at *P*  < 0.05. BER: blossom-end rot.

Within each modality (ungrafted populations; grafted populations), different lowercase letters in the same column indicates significant differences (Tukey test for rootstocks comparisons; t-unpaired test for population comparisons).

Within each population, different capital letters in the same column indicates differences between grafted and ungrafted population at *P* < 0.05 (non-parametric Mann-Whitney U unpaired test).

In the same column, the $ symbol besides the grafted and ungrafted means (averaged for the two populations) indicates differences at *P* < 0.05 (non-parametric Mann-Whitney U unpaired test).

The study of the two grafted populations onto the three commercial rootstocks (King-Kong F1, K; Multifort F1, M; Spirit F1, S) shows that population (P) × rootstock (R) interaction was not significant, which suggests that the behaviour of each of the populations tested was independent of the rootstock used. Additionally, there were no significant differences between the three rootstocks (Table 1). However, the population effect was significant in all cases, Mor-62 showing higher marketable, total yield and mean fruit weight than Mor-204 (averaged for the three rootstocks). As stated for the ungrafted populations, the number of marketable fruits per plant was greater in Mor-204, although in this case the differences were less pronounced. Similarly, BER yield was about seven times higher in grafted Mor-204 than in grafted Mor-62 (0.71 and 0.10 kg pl^−1^, respectively) (Table 1). Analysing each population independently (capital letters in Table 1), grafting increased the marketable and total yield ~50% in Mor-62 and ~30% in Mor-204, while differences in marketable and total fruit number were lower (~40% in Mor-62 and ~10% in Mor-204).

Finally, focusing on the overall effect of grafting on tomato productivity ($ symbol in Table 1), we observe that this technique significantly improved the marketable and total yield (9.71 and 12.1 kg pl^−1^ as average in the grafted plants *vs* 6.77 and 8.34 kg pl^−1^ in the ungrafted ones, respectively).

### Fruit nutritional and functional analysis

For the ungrafted treatments, the only significant differences observed were in the lycopene content (higher in Mor-62) (Table 2). For the grafted populations, there was no significant P × R interaction. The population effect was significant on the glutamic and malic acids, fructose, glucose, ascorbic acid, lycopene and total carotene contents, the highest values corresponding to Mor-62 with except for the ascorbic acid. In relation to the rootstocks, significant differences between them were only noted for the glucose content (the highest values in K; the lowest ones in M). When focusing on each population, the comparison of the grafted and ungrafted treatments (capital letters in Table 2) shows a decrease of reducing sugars and malic and citric acids when grafting, whereas the lycopene and total carotene contents increased with its use in both populations, and also the β-carotene in Mor-62. Additionally, when comparing grafted and ungrafted averages ($ symbol in Table 2), we can see that grafting led to a decrease in the malic and citric acids, and also in the sugar content. On the contrary, the carotenoids increased, in the case of the β-carotene mainly as result of the significant improvement achieved in grafted Mor-62.

**Table 2:** Effect of population, rootstock and grafting on the nutritional and functional composition of fruits of the “Moruno” tomato type.

Treatment	Malic acid (mg·100 g^−1^ fw)	Citric acid (mg·100 g^−1^ fw)	Glutamic acid (mg·100 g^−1^ fw)	Fructose (mg·100 g^−1^ fw)	Glucose (mg·100 g^−1^ fw)	Ascorbic acid (mg·100 g^−1^ fw)	Lycopene (mg·100 g^−1^ fw)	β-carotene (mg·100 g^−1^ fw)	Total carotene (mmol·100 g^−1^ fw)
**Ungrafted**									
Mor-62	212.0 a A	441.5 a A	211.2 a A	2,098 a A	2,184 a A	9.38 a A	6.31 a B	0.76 a B	0.87 a B
Mor-204	189.0 a A	465.2 a A	244.5 a A	2,204 a A	2,244 a A	10.58 a A	4.43 b B	0.90 a A	0.65 a B
Mean	200.5 $	453.4 $	227.9	2,151 $	2,214 $	9.98	5.37 $	0.83 $	0.76 $
**Grafted**									
***Main effects ANOVA***									
Population									
Mor-62	179.2 a B	356.2 a B	217.5 a A	1,791 a B	1,882 a B	9.09 b A	7.83 a A	0.97 a A	1.09 a A
Mor-204	155.9 b B	335.6 a B	184.2 b B	1,558 b B	1,683 b B	11.63 a A	5.71 b A	0.97 a A	0.82 b A
Mean	167.5 $	345.9 $	200.9	1,674 $	1,782 $	10.36	6.77 $	0.97 $	0.96 $
Rootstock									
King-Kong (K)	165.1	367.1	209.6	1,757	1,951 a	10.53	7.06	0.95	0.99
Multifort (M)	162.4	322.9	181.9	1,598	1,598 b	9.24	6.11	0.97	0.87
Spirit (S)	175.1	347.7	211.0	1,668	1,798 ab	11.30	7.14	0.99	1.01
*Significance*									
Population (P)	*	NS	*	*	*	*	*	NS	*
Rootstock (R)	NS	NS	NS	NS	*	NS	NS	NS	NS
P × R	NS	NS	NS	NS	NS	NS	NS	NS	NS
Mor-62									
King-Kong (K)	177.2	388.4	223.1	1,834	2,102	7.87	8.74	0.98	1.21
Multifort (M)	166.1	303.5	179.7	1,654	1,594	8.90	6.32	0.93	0.89
Spirit (S)	194.2	376.8	249.6	1,884	1,950	10.49	8.43	0.99	1.18
Mor-204									
King-Kong (K)	152.8	345.7	196.1	1,680	1,800	13.19	5.38	0.92	0.78
Multifort (M)	158.8	342.4	184.1	1,541	1,602	9.58	5.90	1.01	0.85
Spirit (S)	156.1	318.6	172.5	1,452	1,645	12.10	5.84	0.98	0.84

fw: fresh weight.

NS: not significant; *Significance at *P*  < 0.05.

Within each modality (ungrafted populations; grafted populations), different lowercase letters in the same column indicates significant differences (Tukey test for rootstocks comparisons; t-unpaired test for population comparisons).

Within each population, different capital letters in the same column indicates differences between grafted and ungrafted population at *P*  < 0.05 (non-parametric Mann–Whitney U unpaired test).

In the same column, the $ symbol besides the grafted and ungrafted means (averaged for the two populations) indicates differences at *P* < 0.05 (non-parametric Mann–Whitney U unpaired test).

### Fruit postharvest storage

Ungrafted Mor-62 recorded a higher postharvest storage life (54%), total (∼80%) and averaged daily weight loss (17%) than ungrafted Mor-204 (Table 3). In the case of the grafted populations, as in the previous cases, there was no significant P × R interaction, nor any effect due to the rootstock used. However, the population effect was significant, the highest values corresponding to Mor-62 in all cases (Table 3). Thus, as with the ungrafted populations, fruits from grafted Mor-62, regardless of the rootstock employed, could be stored for a longer time (46%) in refrigerator conditions, doubled the total weight loss and experienced a daily weight loss about 43% higher than grafted Mor-204. However, the comparison of the grafted and ungrafted treatments both in each population (capital letters in Table 3) and averaged across them (lack of $ symbol in Table 3) indicates that grafting had practically no effect on the fruit postharvest behaviour.

**Table 3:** Effect of population, rootstock and grafting on the postharvest storage variables of fruits of the “Moruno” tomato type.

Treatment	Postharvest period (days)	Weight loss (%)	Daily weight loss (%)
**Ungrafted**			
Mor-62	30.1 a A	7.96 a A	0.27 a A
Mor-204	19.5 b A	4.42 b A	0.23 b A
Mean	24.8	6.19	0.25
**Grafted**			
***Main effects ANOVA***			
Population			
Mor-62	25.4 a A	7.49 a A	0.30 a A
Mor-204	17.4 b A	3.52 b A	0.21 b A
Mean	21.4	5.51	0.26
Rootstock			
King-Kong (K)	17.8	5.29	0.29
Multifort (M)	22.6	5.76	0.25
Spirit (S)	23.7	5.47	0.23
*Significance*			
Population (P)	*	*	*
Rootstock (R)	NS	NS	NS
P × R	NS	NS	NS
Mor-62			
King-Kong (K)	20.4	7.26	0.36
Multifort (M)	27.4	8.41	0.31
Spirit (S)	28.4	6.81	0.24
Mor-204			
King-Kong (K)	15.3	3.32	0.23
Multifort (M)	17.9	3.12	0.18
Spirit (S)	19.1	4.13	0.22

NS: not significant; *significance at *P* ≤ 0.05.

Within each modality (ungrafted populations; grafted populations), different lowercase letters in the same column indicates significant differences (Tukey test for rootstocks comparisons; t-unpaired test for population comparisons).

Within each population, different capital letters in the same column indicates differences between grafted and ungrafted population at *P*  < 0.05 (non-parametric Mann–Whitney U unpaired test).

In the same column, the $ symbol besides the grafted and ungrafted means (averaged for the two populations) indicates differences at *P*  < 0.05 (non-parametric Mann–Whitney U unpaired test).

### Sensory analysis

Looking at the ungrafted populations, no significant differences in tomato flavour preference were found (7.28 as average, Table 4). In contrast, a significantly higher rating of the visual appearance of fruits was attributed to ungrafted Mor-62 (8.57 *vs* 6.29 in Mor-204).

**Table 4:** Effect of population, rootstock and grafting on the sensory analysis of fruits of the “Moruno” tomato type.

Treatment	Flavor preference	Visual appearance
**Ungrafted**		
Mor-62	7.00 a A	8.57 a A
Mor-204	7.57 a A	6.29 b A
Mean	7.29 $	7.43
**Grafted**		
***Main effects ANOVA***		
Population		
Mor-62	6.95 a A	7.62 a B
Mor-204	5.90 b B	7.14 a A
Mean	6.43 $	7.38
**Rootstock**		
King-Kong (K)	6.36 ab	7.21
Multifort (M)	5.79 b	7.50
Spirit (S)	7.14 a	7.43
*Significance*		
Population (P)	*	NS
Rootstock (R)	*	NS
P × R	NS	*
Mor-62		
King-Kong (K)	6.86	8.00 ab
Multifort (M)	6.29	8.14 a
Spirit (S)	7.71	6.71 b
Mor-204		
King-Kong (K)	5.86	6.43 b
Multifort (M)	5.29	6.86 b
Spirit (S)	6.57	8.14 a

Values ranked through a hedonic scale from 1 to 9.

NS: not significant; *significance at *P*  < 0.05.

Within each modality (ungrafted populations; grafted populations; rootstocks; rootstocks for each population), different lowercase letters in the same column indicates significant differences; within each population, different capital letters in the same column indicates differences between grafted and ungrafted population; in the same column, the $ symbol besides the grafted and ungrafted means (averaged for the two populations) indicates significant differences. Friedman test (*P*  < 0.05).

According to the grafted treatments, the interaction P × R was not significant for the flavour preference, and the sensory panel preferred grafted Mor-62 rather than Mor-204 (6.95 *vs* 5.90, Table 4) and S rather than M rootstock (7.14 *vs* 5.79). However, P × R was significant for the visual appearance. Thus, M and K rootstocks in Mor-62 led to a very good score for visual appearance (higher than 8.00 on average), while in Mor-204 led to the worst results. The opposite behaviour was obtained for the S rootstock (8.14 in Mor-204 but 6.71 in Mor-62). Analysing each population independently (capital letters, Table 4), this technique did not affect the overall flavour preference in Mor-62, but made it worse in Mor-204 (5.90 *vs* 7.57). Additionally, it improved the visual appearance for Mor-204 (7.14 *vs* 6.29, although the differences were not significant), but worsened it for Mor-62 (7.62 *vs* 8.57). Finally, addressing the overall effect on the sensory traits considered ($ symbol, Table 4), grafting made the flavour preference perceived by the sensory panel worse, mainly due to the negative effect detected for Mor-204.

### Multivariate approach

**Figure 1:**
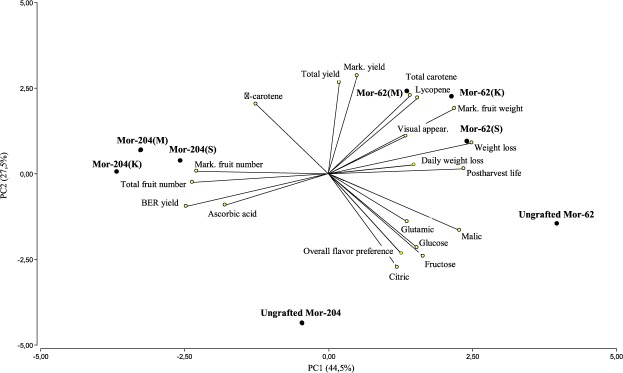
Biplot from the principal component analysis performed with the 19 variables analyzed and the eight treatments (two local “Moruno” [Mor] populations, 204 and 62 ungrafted, and grafted on the three rootstocks: King-Kong F1, K; Multifort F1, M; Spirit F1, S). BER: blossom-end rot. BER yield, marketable and total fruit number, marketable mean fruit weight and postharvest storage variables were very influential on PC1, while marketable and total yield, organic acids (especially malic and citric) and reducing sugars had an influence on PC2. The two populations were clearly differentiated (i.e. Mor-62 on the right and Mor-204, especially when grafted, on the left half of the biplot).

The biplot obtained from the representation of the eight treatments considered in the reference system of PC1 and PC2 (PC: Principal Components; 72% of the total variability) is shown in Fig. 1. BER yield, marketable and total fruit number, marketable mean fruit weight and postharvest storage variables were very influential on PC1 (44.5% of the total variability), corresponding to the longest vectors. Marketable and total yield had an influence on PC2, in addition to organic acids (especially malic and citric) and reducing sugars. Marketable and total fruit number and BER yield were highly positively correlated (angles close to 0° between the corresponding vectors, especially between marketable and total fruit number, with a Pearson correlation coefficient r = 0.94), as well as the marketable and total yield (r = 0.94) and the lycopene and total carotene contents (r = 0.91). High positive correlations were also found between the visual appearance, the marketable mean fruit weight and the lycopene content (r ∼ 0.65). Also reducing sugars, acids (malic, citric and glutamic) and overall flavour preference were also positively correlated (especially glucose and fructose, r = 0.92). BER yield was, on the other hand, negatively correlated with marketable mean fruit weight (r = −0.78) and postharvest storage variables, especially with weight loss (r = −0.71). Also, the marketable and total fruit numbers were negatively correlated with the marketable mean fruit weight and postharvest storage.

In relation to the treatment location, a clear differentiation between the two populations is observed (i.e. Mor-62 on the right and Mor-204, especially when grafted, on the left half of the biplot). Mor-204 was more affected by BER (in concordance with Table 1), produced a higher amount of marketable and total fruits (although they weighed less individually), and showed the poorest results in the fruit postharvest traits. Grafted Mor-62 had the highest marketable and total yield, mean fruit weight, lycopene and total carotene contents. Grafted Mor-204 had the highest BER yield and number of fruits, distant from the variables related to the flavour. The two ungrafted treatments, at the bottom of the biplot, gave the lowest values in the positive direction PC2 variables, especially in marketable and total yield and total carotenoids, but the highest malic and citric acids and sugar contents.

## Discussion

The effect of grafting when using vigorous rootstocks resistant to soil diseases is clear and positive in relation to crop growth and yield in the presence of soil pathogens, but this may not be apparent in the absence of soil-borne diseases or abiotic stress conditions^19^ or when conditions are optimal and therefore plants are supplied with enough resources^20^. In the current experiment, grafting increased marketable and total yield in the two local populations of the “Moruno” tomato type independent on the rootstock in the absence of special stressing conditions, although differences between the populations were observed. This could be explained by a possible enhancement of the photosynthetic area^21^, which was visually observed in this trial, an increase of nutrient uptake^22^, or of water use efficiency^23^. In this study, the increase in marketable yield (43% higher in grafted plants) was derived from both higher number of fruits and higher mean fruit weight (22% and 12%, respectively). Pogonyi *et al*.^24^, however, found higher differences in yield between grafted and ungrafted plants (~62%), mainly caused by the increased fruit weight (45%). Our results are consistent with previous findings when using commercial scions and rootstocks^5,15^ and also with the heirloom tomato “Cuore di Bue” on commercial rootstocks^4^. However, the heirloom scions “Brandywine” and “Flamme” exhibited differential responses to the commercial rootstocks in terms of yield performance, although under nematode infestation^18^. Additionally, under optimal^25^, or even suboptimal growth conditions^21^, grafting did not increase yields in commercial cultivars. In relation to the BER incidence, although grafting can reduce some of the causes which provoke this disorder in fruits (i.e. the uptake of water and nutrients)^26^, we did not observe any significant effect on it, although it is interesting to highlight its low occurrence, particularly in Mor-62. Krumbein and Schwarz^21^ and Schwarz *et al*.^19^, however, when comparing self-grafted tomato plants or grafted onto two commercial rootstocks in suboptimal conditions, found that the rootstocks reduced the incidence of BER significantly.

Sugars and organic acids comprise the majority of the total dry matter content of fresh tomato fruit^27^. Sugars in tomato are mainly the reducing sugars fructose and glucose, generally correlated with the soluble solid content^28^. Citric and malic acids are the major organic acids, with citric predominating^29^. Results from this study indicate that reducing sugars and organic acids decreased to some extent through grafting (with the exception of glutamic acid in Mor-62), as previously stated by Sánchez-Rodríguez *et al*.^30^. These declines were higher in Mor-204 (25%) than in Mor-62 (17%), which corroborates that changes in these variables are barely influenced by the scion. Additionally, the decreased glucose concentration was influenced by both the type of scion and the rootstock employed, as previously reported^21^. It is interesting to note that sugar contents obtained by other authors^1^ are between 1.2% for fructose and 1.4% for glucose, clearly lower than those found in this study. Also, Gonzaléz Cebrino *et al*.^1^ found higher values of these compounds when comparing traditional Spanish tomato varieties, which was attributed, as in the present trial, to both fruits being grown in organic conditions and their ripening state on the plant.

A positive response of organic acid content to grafting was previously found^21^, explained by an enhanced uptake of nutrients such as potassium. This increase, however, was not observed in our trial.

The antioxidant activity of fruit is important to its nutritional and functional value and in maintaining the stability of pigments^31^. In the case of the ascorbic acid content, we did not observe any notable effect from grafting, in agreement with Fernández-García *et al*.^31^. However, Vrcek *et al*.^15^ obtained in commercial varieties a significantly lower content in grafted fruits, explained by the higher plant shoot biomass or vigour and consequently the higher yield per plant. In our study, fruits from Mor-204 had the highest ascorbic acid content, also associated with smaller fruits^32^. The values of this component obtained in the two local populations (10.26 mg 100 g^−1^ as average) are also lower, in agreement with Adalid *et al*.^2^, than the commonly accepted average level in the commercial tomato (20–24 mg 100 g^−1^)^33^. It should be highlighted that the content of this antioxidant depends not only on the genotype, but also on the specific environmental factors during the growing season^2^.

Concerning the carotenoids, lycopene and β-carotene are the tomato carotenes which present the highest nutritional and functional value and provide health benefits. In this study, the results show that, independently of the rootstock employed, grafting increased the carotenoid content in fruits in both populations, with the consequent importance from a nutritional and healthy point of view. However, the literature consulted does not provide a clear pattern for the carotenoid rates when grafting. Thus, Fernández-García *et al*.^31^, with commercial varieties, supported our results; however, Nicoletto *et al*.^17^ observed different behaviours depending on the rootstock/scion combination with landraces. Vreck *et al*.^15^ concluded that these components were not affected by grafting but mainly by the tomato variety, whereas Helyes *et al*.^34^ obtained lower lycopene content in grafted plants, which was explained by the significantly higher yield reached by grafting. This inverse relationship, however, was not observed in our experiment. Fruits from Mor-62 had significant higher lycopene and total carotene contents than Mor-204, also associated with the fruit colour (dark red for Mor-62, brown for Mor-204). In this sense, Roselló *et al*.^35^ demonstrated the high genetic component of these compounds, less dependent on the environment. In both populations, grafting increased the amount of these antioxidants about 25% and also for β-carotene in Mor-62, whereas in Mor-204 this compound experienced a lower increase (8%). Also notable are the high levels of these antioxidants in comparison with those habitually mentioned in the literature, reported as average at 3 and 0.39 mg·100 g^−1^ for lycopene and β-carotene in commercial raw tomatoes, respectively^2^. With traditional varieties, however, values of lycopene higher than 15 mg·100 g^−1^ were obtained^36^, although measured by a different method.

Although previous research on the postharvest study of fruits resulting from grafting was not found, in this study the limited response of the aspects considered (postharvest storage life and weight loss) to grafting is very clear. However, there are marked differences between populations, with Mor-62 showing the highest postharvest period, weight loss over it and, consequently, daily weight loss. It could be related with the fact that Mor-204 was more susceptible to BER, problem associated with calcium-related disorders, even in soils with appropriate levels in this element, which could have led to poorer fruit storage as calcium is one of the main constituents of the cell walls^37^.

Considering the importance of sensory analysis as a measurement of the acceptance of products by consumers, several studies have reported that the levels of reducing sugars (mainly fructose and glucose) and organic acids (mainly citric and malic) in tomato fruits affect not only the taste attributes of sweetness and sourness, but also the overall flavour as perceived by sensory judges^27,28^. Our results also support that flavour could be affected by the malic and citric acids and the fructose and glucose sugars. In addition to the overall organoleptic issues, in the global acceptance of tomatoes by consumers, the external aspects of the fruits (colour and size) are crucial. For this reason, considering the importance of external appearance and increasing attention to the organoleptic properties responsible for flavour, especially with respect to traditional tomatoes, it is interesting to assess whether the grafting technique *per se*, or the rootstock employed, affect the sensory properties of tomato fruits. In this sense, our findings show that grafting and rootstock may have a different effect on the flavour and the overall external appearance rating by the consumers (represented by the sensory panel), hardly depending on the population. On the other hand, the rootstock employed also affected the flavour, with “Multifort F1” (M) the poorest valued; however, regarding the visual appearance, “Spirit F1” (S) reversed the behaviour depending on the scion (the highest valued in Mor-204 but the lowest in Mor-62), thus indicating the importance of the rootstock/scion combination.

The highest decrease in sugars and acids seen in grafted Mor-204 (in comparison with grafted Mor-62) led to a poorer rating for flavour. It would also explain the lower flavour score assigned to the rootstock “Multifort F1”, where the lowest concentrations of these compounds were found, especially of glucose.

Previous studies have also found a strong positive correlation between the ratio of reducing sugars to the amino acid glutamic acid and flavour^38^. In our study, focusing on grafted and ungrafted means (averaged for the two populations), the mentioned ratio decreased from 19.15 (ungrafted) to 17.20 (grafted), and also the overall quality preference decreased from 7.29 to 6.43.

The bigger fruits obtained in grafted Mor-62, together with an intensification of the red colour due to the high lycopene content^39^, might have led to its good visual acceptance. In this context, Di Gioia *et al*.^4^ found that the sensory profiles of fruit were not modified by grafting, although taste panelists expressed a higher preference for purchasing fruits from plants grafted onto “Maxifort F1” due to their overall external aspect and appearance.

In conclusion, we showed that the marketable and total yields, and their components, of the popular, traditional “Moruno” populations grown with organic management, responded very positively to grafting onto commercial rootstocks even in the absence of soil-borne diseases or abiotic stress conditions. However, no incidence on blossom-end rot disorder was observed. The nutritional and functional composition of fruits was also influenced by grafting, leading to an increase in the carotenoid (lycopene, β-carotene and total carotene) content but a decrease in the malic, citric acid and sugar levels. The ascorbic acid content, however, was not affected by this technique, neither were postharvest storage variables. The response of the sensory traits (overall flavour preference and visual appearance) to grafting varied depending on the scion. In general terms, although differences between populations were observed, the lack of interaction population x rootstock should be noted. The multivariate approach considering all the variables and rootstock-scion combinations supported all these findings.

Additionally, more studies including self-grafted treatments as control should be considered for future work in order to increase our knowledge of the “Moruno” type response to grafting.

## Methods

### Field design and culture conditions

Spring-summer field trials were conducted at the experimental farm of the Research Centre “El Chaparrillo” of the Regional Institute for Agro-Food and Forestry Research and Development (3°56′W, 39°0′N, altitude 640 m) located at Ciudad Real (Spain). The climate of this region is continental Mediterranean. The total rainfall and mean temperature during the cropping period were 88.7 mm and 21.2 °C, respectively. The soil was moderately basic (pH_H2O_ 8.3), non-saline (Electrical Conductivity, EC 0.16 dS m^−1^), with a loamy texture, 1.8% of organic matter (Walkley-Black), C/N ratio 10.9, total N 0.08% (Kjeldahl), cation exchange capacity ≈ 20 meq. 100 g^−1^ (ammonium acetate)_,_ available P (Olsen) 49.3 mg kg^−1^, exchangeable K^+^, Mg^++^, Ca^++^, and Na^+^ (ammonium acetate) of 0.9, 2.5, 16.0 and 0.6 meq. 100 g^−1^, respectively, 29.3% of calcium carbonate (Galet) and 10.63% of active calcium carbonate (ammonium oxalate).

The study was developed on two traditional populations of tomato (Mor-62 and Mor-204) belonging to the “Moruno” tomato type, widely grown in Mediterranean countries and greatly appreciated by organic consumers. These populations of indeterminate growth habit are characterized by medium (Mor-204) to large (Mor-62) sized fruits, a dark red (Mor-62) or brown (Mor-204) colour, strong to medium-ribbing intensity, dark shoulders and a predominantly flattened shape. They were grafted onto three vigorous commercial tomato rootstocks with high resistance to several soil diseases [“King-Kong F1” (K), Rijk-Zwaan; “Multifort F1” (M), De Ruiter Seeds; “Spirit F1” (S), Nunhems]. A randomized complete block design with three replicates was chosen. Additionally, ungrafted Mor-62 and Mor-204 were included with three replicates.

Each experimental unit consisted of eight plants spaced 1.0 m apart, with a 2.0 m row spacing (i.e. 5,000 plants ha^−1^). For the different measurements, the central six plants of each plot were considered.

The seeds of the three tomato rootstocks were sown on April 11 in a commercial nursery, two days earlier than the “Moruno” populations. Grafting was done on May 10 by using the spice or tube grafting tool. The grafted and ungrafted nursery seedlings were hand-transplanted in the open air on beds mulched with black plastic 60 micron thick on May 25, using organic farming practices. Fertilization consisted of organic vermicompost (1 kg l.m.^−1^, 2.2% N, 1.5% P and 2.3% K in organic forms), and no chemical fertilizers or pesticides were applied. Irrigation quantities, estimated from the reference evapotranspiration and the phenological stage of the crops^40^, were applied daily by a trickle irrigation system (emitters of 2 l h^−1^ spaced 0.5 m apart, one drip line at the centre of each bed). The total irrigation amount applied was about 5,000 m^3^ ha^−1^. Removal of lateral shoots and basal leaf pruning operations were carried out on plants. The crop cycle lasted 141 days (May 25 to 13 October).

### Trial measurements

Fruits in the optimum ripe stage, when the fruit surface was homogenously red-coloured, were hand-picked through a total of 16 harvests for each treatment (August 3 to 13 October). At each harvest, fruits were classified into marketable or non-marketable portions. In the latter, fruits affected by blossom-end rot (BER), a disorder associated with the uptake and mobility of calcium, more frequent in large-fruit cultivars and under extreme weather conditions^41^, were weighed separately. Marketable and total fruit number and yield were recorded. Additionally, marketable mean fruit weight was calculated from yield and fruit number.

In two consecutive middle harvests (124 and 130 days after transplanting), three healthy fruits per plant (18 fruits per experimental plot) at the ripe stage were taken for the postharvest storage study, the nutritional and functional composition analysis, and the sensory evaluation (i.e., six fruits from each experimental plot for each of the three specific studies per harvest).

For the postharvest storage study, the unwrapped fruits were placed in a controlled cold room simulating the home-refrigerator storage conditions (8 ± 0.5 °C). The duration of the postharvest period (days) of each fruit was judged by appearance (fruits firm to the touch and with commercial visual aspect, before peel becomes soft and shriveled). Additionally, fruits were initially weighed and again at the end of this period, resulting the weight-loss percentage as follows: % Weight loss = [(Wo − Wi)/Wo × 100], where Wo and Wi were the initial and the final fruit weights, respectively. Besides, an overall average estimation of the daily weight loss (%) was calculated as follows: % Daily weight loss = % Weight loss/Postharvest storage life (days).

For the nutritional and functional analysis, fruits were washed with distilled water, homogenized and stored at −80 °C until analysis. In all cases, three analytical replicates per sample were taken.

Reducing sugars (fructose and glucose) and acids (citric, malic, glutamic and ascorbic) were determined by capillary zone electrophoresis using a P/ACE System MDQ (Beckman Instruments, Fullerton, CA, USA). For reducing sugars, citric, malic and glutamic acids, the methodology developed by Roselló *et al*.^42^ was used. For ascorbic acid analysis, the method proposed by Galiana-Balaguer *et al*.^43^ was adopted. For these determinations (reducing sugars and acids), uncoated fused silica capillaries (27 cm total length, 20 cm effective length, 50 µm id) were used for the analytical procedure. Prior to use, new capillaries were rinsed at 50 °C for 5 min with 1 M NaOH, 5 min with 0.1 M NaOH and 10 min with water deionised water. Afterwards, they were rinsed for 30 min with hexadimethrine bromide (HDM), and then with the separation buffer. At the beginning of each measurement, the capillary was rinsed for 30 min with the separation buffer at 20 °C (25 °C for ascorbid acid). Addtionally, for the ascorbic acid determination, before injection in the capillary, all solutions were degassed in an ultrasonic bath and forced through a 0.2 mm membrane filter. Hydrodinamic injection at 3447.38 Pa for 20 s (5 s for ascorbid acid) was used. The detection wavelength was 200 nm for reducing sugars and citric, malic, glutamic acids and 254 nm for ascorbic acid. The separation was performed at −25 kV and 20 °C (−15 kV and 25 °C for ascorbic acid). Between injections, the capillary was rinsed with separation buffer for 5 min. The background electrolyte used for ascorbic acid analysis was 400 mM borate at pH 8 with 0.01% HDM.

Carotenoid (lycopene, β-carotene and total carotene) determination was done by spectrophotometric analysis (Genesys 10 V spectrophotometer, Thermo-Scientific, USA), according to the method described by Roselló *et al*.^35^. The extractions were conducted in the dark to prevent light-induced carotenoid oxidation. Afterwards, 1 mL distilled water was added to separate organic solvent layers and 0.5 mL of the upper layer (hexane phase) was recovered and refrigerated at 4 °C to avoid carotenoid loss. The readings were taken at wavelengths of 510, 503, 452 and 485 nm.

For the sensory analysis, in each of the two harvests considered, the 18 fruits taken for this purpose from each treatment were used to evaluate their overall external appearance (colour and shape preferences) and the overall flavour preference. The sensory analysis was carried out by 13 panelists (professors and students, six males and seven females, aged between 22 and 52) from the Higher Technical School of Agrarian Engineering (University of Castilla-La Mancha, Ciudad Real). The panelists were all familiar with taste panel procedures and the terminology used, and consumed tomatoes at least occasionally. The sensory analysis was performed within the day after harvest, and until then the fruits were stored at ~15 °C. Fruits were washed, cut into wedges and coded with three-digit random numbers. The panelists ranked the fruit samples from the different treatments according to the overall flavour preference and the visual appearance using a hedonic scale from 1 to 9 (1 = low satisfaction; 9 = high satisfaction)^27^. The tests were conducted in partitioned booths in an environment controlled tasting room. Panelists were given water and unsalted crackers between samples.

### Data analysis

In the grafted populations, in order to study the effect of the two factors considered [i.e., population (P) and rootstock (R)] on the analyzed parameters and the possible interaction between them (P × R), a two-way factorial ANOVA was performed. The non-significance of the interaction P × R in most of the analysed parameters allowed the main effects of each factor to be tested. These effects were compared by one-way ANOVA plus a Tukey test for the rootstocks, and an unpaired t-test for the population. In the case of the ungrafted populations, an unpaired t-test was used to compare them. The non-parametric Mann-Whitney U unpaired test was performed to contrast the hypothesis of independence between the parameters analysed and the practice of grafting (grafted/ungrafted), due to the lack of normality. For the sensory analysis, the non-parametric Friedman test was performed to compare the ordinal variables considered (overall flavour preference and visual appearance).

In addition, a graphic multivariate statistical analysis biplot was obtained to show the location of the two populations, ungrafted and grafted on the three rootstocks chosen, in the factorial plane of the two first axes (explaining over 70% of the variance), extracted from the Principal Component Analysis (PCA) performed on the 19 variables analysed (yield and their components, fruit postharvest storage, nutritional and functional composition, sensory analysis), previously standardized^44^.

For the data analysis, the averaged values across the two consecutive sampling dates were considered. All the statistical analyses were performed using Infostat professional v.2017, with a significance level of 0.05 for the mean comparisons.
